# Impacts of the Allocation of Governmental Resources for Improving the Environment. An Empirical Analysis on Developing European Countries

**DOI:** 10.3390/ijerph17082783

**Published:** 2020-04-17

**Authors:** Mihaela Onofrei, Anca-Florentina Gavriluţă (Vatamanu), Ionel Bostan, Bogdan Florin Filip, Claudia Laurența Popescu, Gabriela Jitaru

**Affiliations:** 1 Faculty of Economics and Business Administration, Alexandru Ioan Cuza University, 22 Carol I, 700505 Iasi, Romania; onofrei@uaic.ro (M.O.); bogdan.filip@feaa.uaic.ro (B.F.F.); 2 Faculty of Law and Administrative Sciences, Ştefan cel Mare University, Universitatii 13, 720229 Suceava, Romania; 3 Faculty of Electrical Engineering, Polytechnic University, Splaiul Independenței 313, 060042 Bucharest, Romania; claudia.popescu@upb.ro; 4 Higher Education Finance Department, Ministry of Education and Research, Str. Mendeleev 21−25, Sector 1, 010362 Bucharest, Romania; gabriela.jitaru@uefiscdi.ro

**Keywords:** life expectancy, environment sustainability index, environmental protection public expenses, environmental policies, developing countries

## Abstract

The last years have brought into attention more than ever concerns regarding the accelerating degradation of the environmental conditions which threaten both the present but mainly the future of human society. Under such circumstances, the society and especially the governments need to acknowledge the seriousness of environment issues and be able to develop and put into practice specific policies to counteract them. In this regard, this paper performs an analysis on a group of 11 European developing countries, over the period between 2000 and 2017, aiming first to evaluate the impact of the changing environment conditions and of the governmental actions regarding environment protection, on citizens life, namely on their life expectancy. In order to evaluate, as accurately as possible, the quality of the environment we propose and elaborate upon an environmental sustainability index based on 17 proxies for environment issues, whose values reveal several similarities between some of the analyzed countries. Secondly, using this index and also several categories of public expenses as proxies for the government actions towards environment protection, we analyze their impact on the life expectancy of the citizens during the sample period and draw some specific conclusions on the actions to be taken forward by governments.

## 1. Introduction

The issue of the environment condition which was raised in the past decades of the previous century has become, due to its impacts on individuals and society, one of the major concerns during the latest years. While more and more reports and studies show that, especially in the last decades, the environment condition has aggressively degraded and tends to degrade even faster in the following period, it became obvious that there are needed specific actions to counteract this process. Moreover, many studies have proven a clear connection between the quality of the environment and the health and well-being of the citizens and, therefore, it results also the necessity of actions for ensuring not only stopping the environment degradation but also the improvement of environment condition. The entire human society should be and has already become more and more interested in preserving the environment and for improving its general condition and to take actions towards achieving these goals. This implies that each individual or company has to consider, beyond their income or profit, the impact of its activity on the environment and to find ways for protecting it. However, as history has proven, not all of them are capable of acting like this and this is why, due to the extended public interest on this matter and the substantial financial efforts involved, usually governments are called by people to intervene for solving the environment condition issue.

The latest years have shown that governments from all over the world have acknowledged the importance of the environment condition and proceeded in various ways to find solutions and apply procedures for preserving or improving the environment. Beside imposing laws and regulations for the private sector aiming to protect the environment from pollution, governments also use fiscal instruments in order either to discourage pollutant activities or to encourage green investments. In this regard, governments apply high taxes on activities that generate pollution, but also allocate significant funds for subsidies encouraging people and companies to invest in green energy, electric cars, systems for preventing pollution and so on. 

At the same time, even if taking actions for preserving the environment condition is a necessity, the considerable funds needed for such actions put a significant pressure on the budget of the governments, especially in the case of the developing countries which have to carefully consider their priorities in this area. Therefore, in order to develop and to apply their future environment policies, governments need first to assess, as clearly as possible, the status and the tendency of the environment condition, but also to evaluate the impact of the previous actions made.

Starting from the above considerations, this paper brings into attention an analysis on the impacts of the allocation and investment of the governmental financial resources for improving the environment condition on 11 European developing countries, during 2000–2017 period. As a first part of the contribution to the existing literature, we have built and proposed a new environment condition index, based on 17 specific environment indicators, in order to evaluate more accurately the environment condition status, which may be useful also for the governments’ evaluations on the environment. Secondly, using the environmental sustainability index and several categories of public expenses as proxies for the government actions towards environment protection, we analyze the impact of such factors on life expectancy. To the best of our knowledge, an analysis on European developing countries from this perspective has not been done before and our results may be helpful for governments in order to shape their own policies regarding environmental conditions.

## 2. Literature Review 

The environment condition has been the subject of many studies most of them developed especially after the beginning of the current millennium, on the background of the increased understanding regarding the impact of pollution which generated significant concerns both for the scientists, but also for the common individuals and of the governments. While the rising concerns of the individuals were mostly determined by the impacts of environment degradation on their health and their quality of life, governments and researchers had a much larger perspective looking to determine also specific correlations between environment condition and other economic indicators. In this regard, literature includes studies on the relationships between environment and economic growth [1,2,3,4,5,6,7,8], taxation [9,10,11,12], government or private expenditures [13,14,15,16] in order to find appropriate solutions for counteracting the process of environment’s degradation and to adjust the environmental policies [17,18,19,20,21,22]. 

Moreover, the issue of preserving and improving the environment condition has also been prioritized by international organizations which started, especially in the latest years, to perform more and more studies on the environment evolution, on the potential impacts of macroeconomic or fiscal policies on climate change [23], but also on the reverse impacts determined by climate change on such policies [24] or on the related effects of environmental policies on economic and social life [25]. All such studies have stressed both the accelerated trend of the degradation of the environment and the need for adjusting the macroeconomic and fiscal policies of the governments in order to reduce the negative effects of this process, and to improve the environment condition while mitigating this goal with ensuring economic growth and social welfare. Similar concerns are also subject of studies developed from other researchers [26,27,28,29,30]. 

Starting from the need to preserve the environment in order to avoid the effects of also damaging the public health and life itself, several studies have tried to find solutions for mitigating the purpose of ensuring economic growth with environment protection. Economides and Philippopoulos [31], for instance, have questioned this idea and analyzed the Ramsey second-best optimal economic policy in a general equilibrium model of endogenous growth, augmented with renewable natural resources, concluding that even if an appropriate policy can influence economic growth and environment sustainability, in the long run, this is possible only on the profile of growing economies. They also showed that, in order to mitigate economic growth with the environment protection, governments should base their policy on taxation and on the allocation of tax revenue between infrastructure and cleanup. Moreover, Gupta and Barman [32] have developed a model of endogenous economic growth and analyzed how governments should allocate their tax revenue between pollution abatement expenditure and productive public expenditure and concluded that the optimum income tax rate and the optimum abatement expenditure rate are simultaneously determined with the growth rate in the steady-state equilibrium of their model and sensitive to the pollution rate.

Halkos and Paizanos [33], studied the impact of government spending on the environment on 77 countries for the 1980–2000 period, estimating both the direct effect of government spending on pollution and the indirect effect determined by government spending effect on per capita income and the subsequent effect of income level on pollution, finding that government spending had a negative direct impact on per capita emissions of sulfur dioxide and carbon dioxide, confirming the results of Lopez et al. [34]. They also conclude that a reduction of government expenditure leads to environmental degradation and, therefore, governmental expenditures are needed and also there is a need for the establishment of international environmental treaties.

Furthermore, analyzing the impact of the expenditure of households, companies and government on aggregate and sectoral carbon emissions in the world economies during 1990 to 2015, Adewuyi [14] found that increased public spending has diverse effects on environmental quality at aggregate and sectoral levels, including positive direct effects, but also negative indirect effects in the long term, and therefore pleaded for enhancing industrial and macroeconomic policies to encourage the adoption of low carbon emitting and less energy intensive production technologies. Looking from another perspective, Itaya [35] analyzed the effects of environmental taxation on long-run growth, concluding that environmental taxation generally enhances long-run growth when the balanced growth path is indeterminate, but also harms long-run growth when the balanced growth path is determinate.

The analyses on the environment status and its relationship with different economic or social indicators made in the literature have usually considered only specific aspects of the environment reflected by indicators like sulfur dioxide emissions [33,36], carbon oxide or carbon dioxide emissions [14,37,38], azot dioxide [39], particulate matter [36,39,40], GHG emissions [41], water pollution [42,43] waste and other types of pollutants. However, there are very few studies that are analyzing the entire range of pollutants or that offer a whole image either on the environment condition or on the impacts of all such pollutants on the economic and social life. In this regard, a first attempt to evaluate the environment was made by a consortium of organizations including World Economic Forum’s Global Leaders for Tomorrow Environment Task Force (WEF), Yale Center for Environmental Law and Policy (YCELP), and the Columbia University Center for International Earth Science Information Network (CIESIN) that developed the Environmental Sustainability Index (ESI) [44]. This index, adjusted later, has synthetized the first 22 environmental indicators characterizing five major aspects of environmental sustainability consisting in the state of the environmental system (air, soil, ecosystems and water), the stresses on those systems (pollution and exploitation), the human vulnerability to environmental change (loss of food resources or exposure to environmental diseases), the social and institutional capacity to cope with environmental challenges and the ability to respond to the demands of global stewardship by cooperating in collective efforts to conserve international environmental resources. Later, Wendling et al. [45], developed in a study for the same organizations the Environmental Performance Index (EPI) synthetizing 24 performance indicators across ten issue categories covering environmental health and ecosystem vitality, to evaluate how close countries are to established environmental policy goals. However, even if these indexes combine specific environmental characteristics with impacts on social life and, therefore, offer a straight image on the environment status, an alternative index proposed in this paper is validated by the necessity to make a clear distinction between differing standpoints related to the idea of sustainability and is consolidated by the need to critically evaluate and study the environmental sustainability as a causal framework. Furthermore, the Environmental Sustainability Index (ESI) is computed based on many controversial methods which imply many variables and high financial costs for data collection, revealing why the existent datasets for ESI are before 2005. The Environmental Performance Index (EPI) methodological approach, quantify and numerically marking only the environmental performance of a state’s policies, avoiding the entire causal framework. 

At the same time, it is worthwhile to observe that the issues regarding the environment condition are most of the time linked with the more stressing concerns regarding the impact determined by the environment degradation on the social life, namely on the health of the population. Therefore, several papers [46,47,48,49,50,51] are approaching this subject from different angles, putting accent on how some specific environmental characteristics such as air or climate can determine severe impacts on citizens life, leading even to mortality issues [52]. In this regard, Evans and Smith [53] have also found positive and significant associations between current and long-term exposure to air pollution and the cases of angina or heart attacks which are relevant to air pollution-related mortality.

Other researchers have also studied the impact of the environment condition on people’s well-being but from another point of view. Thus, Brunekreef [54], later Mariani et al. [55] and Jouvet et al. [56] have all analyzed if there is a connection between life expectancy or longevity and the environment. Brunekreef [54] concludes in his study that the impact of small differences in long-term exposure to airborne particulate matter on life expectancy could be substantial. Mariani et al. [55] showed that there is a positive correlation between longevity and environmental quality, both in the long run and along the transition path, concluding that environmental conditions affect life expectancy. 

The above-mentioned studies reveal the importance of environmental sustainability and the subject appears to continue to represent an important goal still to be achieved by researchers. Our study seeks to evaluate the status and the evolution of the environment condition and its impact on people’s well-being. Since the Environmental Sustainability Index (ESI) is computed based on many controversial methods which imply many variables and high financial costs for data collection—with available data before 2005 and the Environmental Performance Index (EPI) quantify and numerically marking only the environmental performance of a state’s policies, avoiding the entire causal framework—we first evaluate the quality of the environment by using Factor Analysis, a method which avoids the problems of skewed distribution and it reduces the costs of data collection. Secondly, using this index and also several categories of public expenses as proxies for the government actions towards environment protection, we analyze their impact on the wellbeing of EU developing countries’ citizens and draw some specific conclusions on the actions to be taken forward by governments.

The results sustain a strong positive relationship between governmental actions regarding environment protection (measured by the sustainability index and the different categories of public spending) and population health, being in line with studies which confirm that life expectancy and environmental quality dynamics are jointly determined, and confirms the need of adjusting the macroeconomic and fiscal policies in order to preserve the environment and to avoid the effects of damaging the public health and life itself.

We contribute to the literature by establishing the relationship between a large set of adopted measures on the area of environmental sustainability and wellbeing of EU developing countries’ citizens. Further, we contribute in terms of countries included in our analysis, the period and input variables used to assess the relationship between the environment condition, public expenses and life expectancy. 

## 3. Materials and Methods

The accelerated degradation of the environmental conditions which threaten both the present but mainly the future of human society, requires proactivity and strategic policies to counteract them. The environment plan should be part of government plans and it is important to set out the goals for improving the environment within a generation and leaving it in a better state. By imposing laws and regulations for the private sector aiming to protect the environment from pollution, governments use fiscal instruments in order either to discourage pollutant activities or to encourage green investments. However, despite multiple pathways for improvements of environmental quality at the EU level, socio-economic and cultural context still guide the major differences in human health between European countries. On the profile of developing countries, even if the environmental problems have received political recognition, poverty becomes a vector associated with hazards related to pollution and other environmental diseases [57,58,59]. Our analysis combines the ordinary least-squares regression model (OLS) analysis with factor analysis and tests this on a group of 11 European developing countries, over the period from 2000 to 2017, to assess the impact of the changing environmental conditions, governmental actions regarding environment protection, and the impact on citizens’ lives. In order to accurately evaluate the quality of the environment, we propose and elaborate an environmental sustainability index based on 17 proxies for the environmental issues, whose values reveal several similarities between some of the analyzed countries. Secondly, using this index and also several categories of public expenses as proxies for the government actions towards environment protection, we analyze their impact on the life expectancy of the citizens during the sample period and draw some specific conclusions on the actions to be taken forward by governments.

Based on the study of Yan and Hauschilda [60], which analyzes environmental sustainability indicators and categorizes them into Drivers-Pressures-Response-States-Impacts, we establish the components of the environmental sustainability index (INDEX_env). Thus, one of the most important consumers of liquid fuels is represented by the transportation sector, which is reflected in its contribution to air pollution and Greenhouse gas (GHG) emissions in tons/per capita (GHC_T), Emissions of nitrogen oxides kg/capita (NOx), Emissions of Volatile organic compounds kg/ capita (VOC), Carbon monoxide emissions kg/ capita (CO) and Emissions of sulfur oxides kg/ capita (Sox). Therefore, the annual GHC emission due to the transportation sector and the air pollution indicators (such as NOx, VOC, Sox) indicate the “pressures” on the environment.

Improvement in area of waste management, in particular by reducing landfilling and increasing separate collection of waste in the southern regions, can influence municipal waste generation (kg/cap) and can increase pollution, grime or other environmental problems, which allows us to select these indicators based on the fact that we can consider them the “drivers”, because they conduct to human activities that negatively affect the environment and cause a type of “pressures” (W_tr, MW_tr, POL). On the other hand, as a result of the pressures, the “state” of the environment is changed and this may cause an “impact” on the environment, which may eventually trigger a political “response”. This response can consist in environment taxes, economic instruments to address environmental problems, which are designed to internalize environmental problems and to ecologically sustainable activities. In our case, it is represented by Env_tax, Poll_Tax and Transp_Tax, which means environmental tax (% from taxes and social contributions), Pollution taxes (% GDP) and Transport taxes (% GDP), respectively. 

The last indicators which are used in computation of environment sustainability index are classified as impact and state indicators and are related to human health damage and ecosystem quality. In respect to this, even if the most commonly used metric to indicate damage to human health is DALYs, per thousand inhabitants Ambient Particulate Matter (DPTI), because it expresses the reduced quality of life due to illness in years as well, given the multiple causes of human health damage, the literature validates the implication of the variables used below [61,62,63]. More exactly, we talk about Premature deaths/million inhabitants Ambient Particulate Matter (Pre_deathinh), Premature deaths, % total premature deaths Ambient Particulate Matter (Pre_deathT), Welfare cost of premature deaths, % GDP equivalent Ambient Particulate Matter (WCPD), Value of a statistical life, millions national currency current prices Ambient Particulate Matter (VOSL), DALYs, per thousand inhabitants Ambient Particulate Matter (DPTI) and Carbon dioxide emissions tons per capita (CO2_T). 

To estimate the index of environment sustainability status, we performed factor analysis, a non-parametric analysis widely used as an effective method for the construction of composite indicators, which according to Samita and Abeynayake [64] is a method helpful for analyzing and exploring the design of the sub-indicator sets. Therefore, it is obvious that the method helps us to discern the factors which have significant contribution to environment sustainability. Mathematical eloquence of factor analysis is the orthogonal-linear transposing data in a system of coordinates so that the greatest variance of projection data becomes the first coordinated, and the second largest variance becomes the second main component; that is why, in the results, each variable is given a ‘uniqueness’ score, and the first factors explain the percentage of total variance. The method offers the advantage of generating independent components, in contrast to the analysis of the main components—excluding the orthogonal relationship between components, and following the literature insights—and has a greater capacity to capture components (Wang et al., 2009) [65]. Following the methodological approach, we first calculated the weighted average of the three factors and then we estimated mathematically by taking into account the percentage of variance for each factor (see Equation (1)), and then, following the Equation (2), we apply a normalization procedure validated in the study performed by Eck and Waltman [66].

(1) $$M=\frac{\sum_{t=1}^{n}W_{t}*V_{t}}{\sum_{t=1}^{n}W_{t}}$$

(2) $$Z_{ij}=\frac{x_{ij-{\bar{x}}_{j}}}{s_{j}}$$

*M* = average value
*V* = actual value
*W* = Weighting factor
*N* = number of periods in the weighting group
*X_ij_* = Data for variable *j* in sample unit *i*
${\bar{x}}_{j}$ = Sample mean for variable *j*
*s_j_* = Sample standard deviation for variable *j*

Given that our work contributes to the existing literature based on period and input variables used to assess the relationship between the environment condition, public expenses and life expectancy, it requires new methodological insights in the process of identified EU developing countries with a positive (negative) directional indicator of environmental sustainability. More specifically—even if the actual theoretical framework reveals the existence of the Environmental Sustainability Index (ESI), which is a measure of overall progress towards environmental sustainability, and the Environmental Performance Index (EPI), which is focused on a core set of environmental outcomes linked to policy goals—due to data unavailability and the use of controversial methods which imply many variables and high financial costs for data collection, we measured the status of environment conditions by using Factor Analysis, a method which avoids the problems of skewed distribution and it reduces the costs of data collection.

Besides the fact that the Environmental Sustainability Index is computed based on many controversial methods which imply many variables and high financial costs for data collection, with available data before 2005, the literature insights highlight also the dilemmas regarding the structure and methodology of the ESI, such as problems of cause and effect or weighting problems, and invoke that there is not yet a completely satisfactory index of environment sustainability [67,68]. Regarding the Environmental Performance Index (EPI), it can be observed an important contribution on quantify and numerically marking only the environmental performance of a state’s policies, but it avoids the entire causal framework, being also criticized from the point that, based on methodological approach, it allows each evaluated country to look for its own optimal weights that maximize the composite indicator relative to the other countries [69]. However, our work does not contest the significant effort regarding the computation of the Environmental Sustainability Index and Environmental Performance Index; we just highlight the usefulness of our methodological approach and we justify the proposal for an alternative path to identify the status of EU developing countries with a positive (negative) directional indicator of environmental sustainability. That is why we apply Factor Analysis, a method which offers the advantage of generating independent components, exclude the orthogonal relationship between components, avoids the problems of skewed distribution and has a greater capacity to capture the basic components of environment conditions. 

After we establish the status of environment conditions, in order to assess the implication of environment sustainability on life expectancy, we use an ordinary least-squares regression model (OLS) analysis. The model includes relevant explanatory variables that influence the level life expectancy, several categories of public expenses as proxies for the government actions towards environment protection and the index of environment sustainability:

(3) $$Life\_expec{\;}_{it}=c_{0}+c_{1}\times Env\;expT_{i,t}+c_{2}\times Env\_exp\_GDPE_{i,t}+c_{3}\times Env_{ex}P_{i,t}+\;c_{4}\times CHEXP_{i,t}+c_{5}\times DGEXPT_{i,t}+c_{6}\times EPEXPG_{i,t}+c_{7}\times WMNGE_{i,t}+c_{8}\times \;Env\_protectionTexp_{i,t}+c_{9}\times INDEX\_envS_{i,t}+u_{i,t}$$

where *i* and *t* indicate the country and year for each variable. The dependent variable $\mathrm{Life}\_{\mathrm{expec}\;}_{\mathrm{it}}$ represent a key metric for assessing population health and indicates Life expectancy at birth, total (years). The independent variables and the components of environmental sustainability index are presented in Appendix A Table A1. The list of the examined countries includes Bulgaria, Croatia, Czech Republic, Estonia, Hungary, Latvia, Lithuania, Poland, Romania, Slovakia, Slovenia.

The literature points out the relationship between social and environmental factors and life expectancy, finding a linear relationship between the abovementioned variables and health care associated factors, validating the fact that the health system requires policy and programs targeted to improve environment sustainability [70,71,72,73]. Concerning the implication of governmental actions regarding environment protection on citizens’ lives, the literature insights examine life expectancy and infant mortality as the ‘output’ of the health care system, and some environmental and occupational factors [74,75]. The environmental sustainability indicators which compute the environmental sustainability index (INDEX_env) are validated by the study of Yan and Hauschilda [60], which categorizes them into Drivers-Pressures-Response-States. 

Based on the rationale that panel data approach offers more efficiency in estimation, we used two different modeling strategies. As a first step in the analysis, the study used a pooled ordinary least-squares regression model (OLS) as a benchmark. Secondly, we draw on a panel fixed effects (FE) model. The approach can be explained first by the fact that through this method we control the so-called unobservable constant heterogeneity and consider the peculiar characteristics of each of the countries. Second, based on the fact that our panel have some omitted variables, the method used controls for endogeneity generated by omission of explanatory variables. The equation for fixed-effects model has the following form:

(4) $$Y_{i,t}=\alpha_{i}+X_{i,t}\times \beta +\varepsilon_{i,t,}$$

in which $Y_{i,t}$ is the dependent variable observed for country *i* at time *t*, *X_i,t_* is the time-variant regressor matrix, $\alpha_{i}$ represents an unknown country-specific constant (the “fixed effect”) and *ε_i,t_* is the error term. To determine the appropriateness of the fixed-effects panel data estimation, we perform a Hausman test and we check fixed effects (FE) vs random effects (RE). The results of the Hausman test (available at results part) reveal that the fixed-effect model was to be used.

## 4. Results and Discussion

In this section, we present the main findings of the research. Table 1 presents the results from the factor analysis, aiming to reveal the factors which have an important contribution to environmental sustainability and compute the composite index of environment sustainability. Table 2 reports the estimation based on pooled OLS, random- and fixed-effect panel data model. To validate the model used, we control for autocorrelation, heteroscedasticity, and serial correlation by using the Driscoll–Kraay model with the robust covariance matrix. The problems of spurious regression were solved by using logarithmically transformed variables in regression analysis. To eliminate the multicollinearity problems, we calculated the VIF test and, given that the results show values below three, we establish that there are no multicollinearity problems for our models. To control the so-called unobservable constant heterogeneity and to solve the problem of some omitted variables, the study used a pooled ordinary least-squares regression model (OLS) as a benchmark and then report the results for panel fixed effects (FE) model.

By using annual data for the 2000–2017 period, the study indicated that the three main factors obtained explain the evolution of the variables used. More exactly, factor 1, 2 and 3 have explained over 90% of the total variance (See Table 2). To estimate the index of environment sustainability, we first calculated the weighted average of the three factors, and we estimated mathematically by taking into account the percentage of variance for each factor. As a final step, we apply a normalization procedure indicated by Ansari et al. (2013) and Eck and Waltman [66,76] in line with Equation (1) and (2).

Even if the indicators for environment sustainability index were calculated for 18 years reference and they were integrated into our OLS model, considering the required judgment of approach validation, we exemplify in Table 3 the results for some key cycles of economic conjuncture. Thus, we reported the results for 2006 and 2009, because the first year is characterized by start vulnerabilities that developed in the financial system and on the profile of 2009 we talk about a worst economic downturn, which means that, without proactive governmental policies, the status of environmental conditions can be radically changed. The reported values for 2015 and 2019, reveal the status of developing countries with a positive directional indicator of environmental sustainability (+) in post crisis period. Besides that, we split our results in three groups of countries with specific characteristics: countries with a positive directional indicator of environmental sustainability (+) post crisis period, countries with a negative directional indicator of environmental sustainability (−) in all the period and countries with a positive directional indicator of environmental sustainability (+) in a crisis period. The units are represented by the output values regarding environmental sustainability in EU developing countries (Positive Directional Indicator of environment sustainability (+)/Negative Directional Indicator of environment sustainability (−). From the path analysis results, the study confirms that the environmental deterioration after the financial crisis, social determinants of health, the food crisis, climate change and all environmental problems seem to hamper policy-makers to be assertive in consolidating proactive public spending plans in the area of environment protection. Following the literature insights, Liu and Peter [77] have found that the global financial crisis has created more domestic demand for consumption and implement massive infrastructure construction, which leads to consequent environmental challenges and major problems concerning environmental pollution and resource scarcity. 

The retrospective on environment conditions in EU developing countries reveal the relationship between the environmental problems and policies, being clear that the low results for environment conditions are reported due to the incapacity of policy makers to act proactively in different stages of the economic cycle. Our results indicate that some EU developing countries such as Romania, Estonia and Slovenia, recorded negative directional indicators of environmental sustainability (−) in all the period and reveal environmental deterioration after the financial crisis on the profile of most of EU developing countries, except the Visegrád Group and Croatia. It seems that the results on the profile of the Visegrád Group are supported by coordination mechanisms and proactive policy which allow these countries to work as in alliance, to support each other’s policies and to use common resources in a more sustainable way [78]. Moreover, the positive results can be explained by the environmental management system (EMS) from these countries, which according to Urbaniec [79], strongly supports good practices and solutions in the field of environment management. It should be noted that the Visegrád Group share a common historical and economic heritage and, in recent years, have implemented the concept of sustainable development in sectoral policies and companies. The common cooperation frameworks that these countries are addressing contributed to environmental sustainability and helped to pass the difficulties experienced as a result of the financial crisis. An example can be the water management plans of the border river basins of the Tisza and the Bodrog and cross-border cooperation projects which consolidate between Hungary and Slovakia, a better achievement of the main objective of the EU Water Framework Directive.

After we have evaluated as accurately as possible the quality of the environment through the calculation of the index of environmental sustainability, in order to test the impact of the changing environmental conditions and of the governmental actions regarding environment protection on citizens life, we used this index and several categories of public expenses as proxies for the government actions towards environment protection, and we analyzed their impact on the life expectancy of the citizens. The main empirical results are presented in Table 4, were we estimated our model specification using the estimation based on pooled OLS, random- and fixed-effect panel data model. In column two, we reported the results for pooled ordinary least-squares regression model (OLS), column four indicates the results for panel fixed effects (FE) model. To avoid the implication of spurious regression or the existing non-linear relationship between the independent and dependent variables, and to achieve a normal distribution of variables, some of the variables were logarithmically transformed (see Table A2). With reference to life expectancy at birth, it is highlighted that the minimum age, 70.2, was reported in Latvia in 2002 and the higher value in Slovenia in 2016 and 2017 (81.2). The status of environmental conditions, measured by the environmental sustainability index, indicate the higher directional indicator of environmental sustainability on the profile of one of the Visegrád Group countries, Czech Republic, which recorded in 2000 a level of 2.45056. The lowest level was reported in Slovenia, with a value of -1.705149 in 2011, being among the countries with a negative directional indicator of environmental sustainability (−) in the whole period (see Table 4). Regarding the total general government expenditure for Environmental protection, it seems that Estonia reported the lowest level in 2011, a value of −47 million euro, being a country with negative total expenditure on environment protection. The negative values in 2010 and 2011 are explained by the non-financial non-produced assets, i.e., sales of AAUs, which were recorded as negative values. The sales were 180 million euros in 2010 and 185 million euros in 2011 according to the Environmental Investment Centre (EIC) yearbook, 2011 (EIC 2012). This situation also persists on the values of Environment expenditure as a percent from GDP and on Public Expenditure, with Environment % of total Public Expenditure, when Estonia recorded in the same year negative values (-0.8% for Env_exP in 2010); it is also reported on the profile of Czech Republic, which recorded in 2009 and 2010 negative values for Environmental protection n.e.c., Total gov exp (mil. EUR), −0.3 million euro in 2009 and, for Total environmental protection not elsewhere classified, the expenditure for Environmental protection as a percentage of total general government expenditure was -0.6 % in the same year. Poland scored the highest values regarding total general government expenditure for Environmental protection, 2669.8 million euros in 2010. The lowest level of Current health expenditure (% of GDP) was recorded in Romania in 2000 (4.187934) and the highest in Slovenia in 2013 (8.792162% of GDP).

These results show that almost all coefficients are statistically significant and in a positive relationship with human health. It can be observed that in European developing countries, over the period between 2000 and 2017, life expectancy and environmental quality dynamics are jointly determined. 

The coefficients for the panel fixed effects (FE) model indicate that there is a positive and significant relationship between the environmental sustainability index, which was measured using Drivers-Pressures-Response-States-Impacts indicators, and human health. This can be restated as follows: in EU developing countries characterized by good environmental conditions and of the sustainable governmental actions regarding environment protection, the population has higher health conditions and life expectancy increases. The results are in line with the results highlighted in a study of Mariani et al. [55], which showed that there is a positive correlation between longevity and environmental quality, both in the long run and along the transition path, concluding that environmental conditions affect life expectancy.

Regarding the relationship between human health and government actions towards environment protection (measured by different categories of public spending), the results show that some categories of expenditures directly influence life expectancy. A higher level of environmental spending such as Total environment expenditure (Env_expT), Domestic general government health expenditure (DGEXPT) and Total environmental protection not elsewhere classified expenditure for Environmental protection—as percentage of total general government expenditure (Env_protectionTexp)—will increase the quality of human health. These results are in line with Halkos and Paizanos’ [33] research, which concludes that a reduction of government expenditure leads to environmental degradation, and consolidates Adewuyi [14] results, which state that increased public spending has diverse effects on environmental quality at aggregate and sectoral levels, including positive direct effects. Regarding the relationship between Current health expenditure (CHEXP) and life expectancy, since the indicator includes only healthcare goods and services consumed during each year and exclude capital health expenditures such as buildings, machinery, IT and stocks of vaccines for an emergency or outbreaks, it is found to negatively impact human well-being. In other words, spending the money without taking care of what capital health expenditures means, without being proactive and buildings machinery or stocks of vaccines for an emergency, you cannot positively impact human well-being because you are not focused on saving human lives. 

## 5. Conclusions

The present paper combines ordinary least-squares regression model (OLS) analysis with factor analysis and tests on a group of 11 European developing countries, over the period between 2000 and 2017, the impact of the changing environmental conditions and of the governmental actions regarding environment protection on citizens’ lives. There are some ways in which the paper brings additional insights to research on this area. First, in order to evaluate the quality of the environment, we propose and elaborate an environmental sustainability index based on 17 proxies for the environmental issues, whose components were categorized into Drivers-Pressures-Response-States-Impacts. Secondly, using this index and several categories of public expenses as proxies for the government actions towards environment protection, we analyze their impact on the life expectancy of the citizens during the sample period. The results confirm the literature insights and reveal that the global financial crisis leads to consequent environmental challenges and major problems concerning environmental pollution and resource scarcity. 

Our findings bring into focus some interesting features regarding the EU developing countries’ environment conditions and indicate that countries such as Romania, Estonia and Slovenia, recorded negative directional indicator of environmental sustainability (−) in the whole period. Environmental deterioration persists after the financial crisis on the profile of most EU developing countries, except the Visegrád Group and Croatia. The results on the profile of the Visegrád Group indicate that the environmental management system (EMS) from these countries strongly supports good practices and solutions in the field of environmental management and can be a result of common historical and economic heritage that, in recent years, have implemented the concept of sustainable development in sectoral policies and companies. When considering the relationship between environment condition and life expectancy, we found a strong positive relationship between governmental actions regarding environment protection (measured by sustainability index and different categories of public spending) and population health. Therefore, the results proved to be in line with studies which confirm that life expectancy and environmental quality dynamics are jointly determined and confirms the need to adjust the macroeconomic and fiscal policies of the governments in order to preserve the environment and to avoid the effects of damaging the public health and life itself. 

In line with the paper findings, the entire retrospective of this work allows us to exemplify some policy suggestions and implications in the frame of European Environmental Policy. First, considering that the examined countries are the Member States and the Visegrad Group countries reported positive values of environment conditions, it can be useful to strengthen procedures for defining common policy objectives. Second, the EU decision-makers must deal with coordination mechanisms and proactive policy tools which allow the member countries to work in alliance to support each other’s policies and to use common resources in a more sustainable way.

## Figures and Tables

**Table 1:** The results of factorial analysis of the main components for estimating the environment sustainability index.

Factor Analysis/Correlation, Method: Principal Factors, Rotation: (Unrotated)				
Factor	Eigenvalue	Difference	Proportion	Cumulative
Factor1	4.80030	1.64136	0.3602	0.3602
Factor2	3.15894	1.55483	0.2370	0.5972
Factor3	1.60410	0.34046	0.1204	0.7176
Factor4	1.26364	0.17982	0.0948	0.8124
Factor5	1.08383	0.23232	0.0813	0.8938
Factor6	0.85151	0.28383	0.0639	0.9577
Factor7	0.56769	0.39386	0.0426	1.0003
Factor8	0.17383	0.05780	0.0130	1.0133
Factor9	0.11603	0.05761	0.0087	1.0220
Factor10	0.05842	0.02698	0.0044	1.0264
Factor11	0.03143	0.03485	−0.0024	1.0287
Factor12	−0.00341	0.00724	−0.0003	1.0285
Factor13	−0.01066	0.03648	−0.0008	1.0277
Factor14	−0.04714	0.00294	−0.0035	1.0242
Factor15	−0.05008	0.06249	−0.0038	1.0204
Factor16	−0.11257	0.04669	−0.0084	1.0120
Factor17	−0.15926	.	−0.0120	1.0000

Source: Authors’ calculation.

**Table 2:** Factor Loading and Explained variance.

Unrotated Loadings				
F1	F2	F3	Uniqueness
GHG_T	0.6949	0.4874	−0.0404	0.2780
CO	0.6955	0.5469	0.0075	0.2172
Nox	0.7690	0.5115	−0.1492	0.1247
Sox	0.7250	0.3676	−0.2558	0.2738
VOC	0.3004	0.7201	0.0142	0.3910
W_tr	0.0299	0.1848	0.4907	0.7242
MW_tr	−0.1755	−0.1989	−0.1856	0.8952
POL	−0.3009	−0.2039	0.3365	0.7546
Enx_tax	−0.0592	0.2046	0.3589	0.8258
Poll_tax	0.0432	0.1976	0.7060	0.4607
Transp_Tax	−0.0882	0.2221	0.2221	0.8045
Pre_deathinh	−0.7371	0.6548	0.0201	0.0275
Pre_deathT	−0.5036	0.2867	−0.5912	0.3147
WCPD	−0.7227	0.6684	0.0480	0.0287
VOSL	−0.2731	−0.2818	0.0101	0.8459
DPTI	−0.7306	0.6487	−0.0289	0.0445
CO2T	0.7507	−0.0585	0.0855	0.4257
Factor	Variance	Difference	Proportion	Cumulative
F1	3.97941	0.04346	0.2986	0.2986
F2	3.93595	2.28796	0.2953	0.5940
F3	1.64799	---	0.1237	0.7176

Source: Authors’ calculation.

**Table 3:** The evolution of the environmental sustainability index in the post-crisis and ante crisis period for EU developing countries.

Countries With Positive Directional Indicator of Environmental Sustainability (+) Post Crisis Period								Countries With Negative Directional Indicator of Environmental Sustainability (−) in All the Period						
(+)	CZ	HU	SK	PL	HR				(−)	EE	RO	SI		
2006	−0.09	1.83	0.60	−0.08	1.31				2006	−0.42	−0.22	−0.99		
2009	1.92	−1.21	−0.95	1.06	1.01				2009	−0.86	−0.65	−0.23		
2015	0.03	0.04	0.49	0.97	0.98				2015	1.41	−0.80	−0.21		
2017	0.12	1.42	0.73	0.11	1.34				2017	−0.39	−0.41	−0.24		
Countries with positive directional indicator of environmental sustainability (+) in crisis period														
					(−+−)	LT	LV	BG						
					2006	−1.41	−0.92	−0.95						
					2009	0.04	0.22	0.07						
					2015	−0.25	−0.92	−0.13						
					2017	−1.43	−1.07	−9.34						

Source: Authors’ calculation.

**Table 4:** Main empirical results.

Independent Variables	Dependent Variable- Population Health (Life_Expec)		
	Pooled OLS	Random Effect	Fixed Effect
Env_expT	0.008* (0.006)	0.012* (0.049)	0.012** (0.032)
Env_exp_GDPE	0.024 (0.042)	−0.021 (0.033)	0.020 (0.026)
Env_exP	−0.018 (0.033)	0.008 (0.028)	−0.028 (0.026)
CHEXP	−0.010 (0.024)	−0.033 (0.019)	−0.065** (0.012)
DGEXPT	0.081** (0.028)	0.147** (0.018)	0.165** (0.014)
EPEXPG	−0.015 (0.015)	−0.011 (0.008)	−0.011 (0.084)
WMNGE	0.005 (0.006)	−0.003 (0.040)	−0.001 (0.027)
Env_protectionTexp	0.008 (0.018)	0.015 (0.078)	0.021** (0.065)
INDEX_envS	0.004 (0.033)	0.005 (0.028)	0.007* (0.023)
Cons	4.165** (0.069)	4.095** (0.056)	4.175** (0.418)
F	6.90***	200.56***	141.91***
Hausman		31.94***	
N	155	154	154
R^2^	0.58	0.62	0.52

Notes: Standard errors are reported in parentheses. “*”, “**” and “***” denote statistical significance at 10%, 5%, and 1%, respectively.

## Appendix A

**Table A1:** Description of the variables employed in the analysis.

Name	Code	Source	Definition
**Variables employed in the regression analysis**			
Life expectancy	Life_expec	Eurostat Database	Life expectancy at birth indicates the number of years a newborn infant would live if prevailing patterns of mortality at the time of its birth were to stay the same throughout its life.
Total environment expenditure	Env expT	Eurostat Database	Total general government expenditure for Environmental protection in millions of Euro
Environment expenditure % GDP	Env_exp_GDPE	Eurostat Database	Total general government expenditure for environmental protection as percentage of GDP
Public Expenditure with Environment% of total Public Expenditure	Env_exP	Eurostat Database	Total general government expenditure for environmental protection as percentage of total general government expenditure
Current health expenditure (% of GDP)	CHEXP	World Bank Database	Level of current health expenditure expressed as a percentage of GDP. The indicator includes healthcare goods and services consumed during each year and except capital health expenditures such as buildings, machinery, IT and stocks of vaccines for emergency or outbreaks.
Domestic general government health expenditure (% of GDP)	DGEXPT	World Bank Database	Public expenditure on health from domestic sources as a share of the economy as measured by GDP.
Environmental protection n.e.c. Total gov exp (mil. EUR)	EPEXPG	Eurostat Database	Total environmental protection not elsewhere classified expenditure for Environmental protection in millions of Euro
Waste Management Total gov exp (%GDP)	WMNGE	Eurostat Database	Total waste management expenditure for Environmental protection as percentage of total general government expenditure
Environmental protection n.e.c. Total gov exp (%of total exp)	Env_protectionTexp	Eurostat Database	Total environmental protection not elsewhere classified expenditure for Environmental protection as percentage of total general government expenditure
**Variables employed in the factor analysis** $(\mathrm{Environmental}\;\mathrm{sustainability}\;\mathrm{Index}:\;INDEX\_envS_{it}$ **)**			
Greenhouse gas (GHG) emissions tons/ per capita	GHG_T	OECD.Stat database https://stats.oecd.org/	Greenhouse gas (GHG) emissions tons/ per capita
Carbon monoxide emissions kg/ capita	CO	OECD.Stat database https://stats.oecd.org/	Carbon monoxide emissions kg/ capita
Emissions of nitrogen oxides kg/ capita	Nox	OECD.Stat database https://stats.oecd.org/	Emissions of nitrogen oxides kg/ capita
Emissions of sulphur oxides kg/ capita	Sox	OECD.Stat database https://stats.oecd.org/	Emissions of sulphur oxides kg/ capita
Emissions of Volatile organic compounds kg/ capita	VOC	OECD.Stat database https://stats.oecd.org/	Emissions of Volatile organic compounds kg/ capita
Waste Water treatment %	W_tr	OECD.Stat database https://stats.oecd.org/	Waste Water treatment %
Municipal Waste kg/capita	MW_tr	OECD.Stat database https://stats.oecd.org/	Municipal Waste kg/capita
Pollution, grime or other environmental problems (% population)	POL	Eurostat Database	Percentage of population affected by pollution, grime or other environmental problems
Environmental taxes % of total taxes and social contributions	Enx_tax	Eurostat Database	Total environmental taxes as percentage of total taxes and social contributions
Pollution taxes (% of GDP)	Poll_tax	Eurostat Database	Total pollution taxes as percentage of GDP
Transport taxes (% of GDP)	Transp_Tax	Eurostat Database	Transport taxes as percentage of GDP
Premature deaths/ million inhabitants Ambient Particulate Matter	Pre_deathinh	OECD.Stat database https://stats.oecd.org/	Premature deaths per 1million inhabitants caused by Ambient Particulate Matter
Premature deaths, % total premature deaths Ambient Particulate Matter	Pre_deathT	OECD.Stat database https://stats.oecd.org/	Premature death as percentage of total premature deaths caused by Ambient Particulate Matter
Welfare cost of premature deaths, % GDP equivalent Ambient Particulate Matter	WCPD	OECD.Stat database https://stats.oecd.org/	Welfare cost of premature deaths as percentage GDP equivalent caused by Ambient Particulate Matter
Value of a statistical life, millions national currency current prices Ambient Particulate Matter	VOSL	OECD.Stat database https://stats.oecd.org/	Value of a statistical life in millions national currency current prices related to Ambient Particulate Matter
DALYs, per thousand inhabitants Ambient Particulate Matter	DPTI	OECD.Stat database https://stats.oecd.org/	Number of lost years of "healthy" life per thousand inhabitants caused by Ambient Particulate Matter
Carbon dioxide emissions tons per capita	CO2T	OECD.Stat database https://stats.oecd.org/	Carbon dioxide emissions tons per capita

**Table A2:** Descriptive statistics.

Variable	Mean	Std. Dev.	Minimum	Maximum	Skewness	Kurtosis
Life_expec	75.0298	2.45319	70.2	81.2	0.20	1.79
INDEX_envS	−0.0188653	0.9863578	−1.705149	2.45056	0.40	2.28
Env_expT	565.8263	619.2762	−47	2669.8	0.62	2.61
Env_exp_GDPE	0.6974747	0.2632165	−0.3	1.4	1.68	5.16
CHEXP	6.576177	1.034772	4.187934	8.792162	−0.31	4.11
DGEXPT	4.695633	0.9362482	2.735813	6.75997	0.044	2.28
EPEXPG	0.1333333	0.1518081	−0.3	1.1	0.15	2.12
WMNGE	0.5934343	0.441855	0.1	2	3.16	19.4
Env_protection_Texp	0.3191919	0.3691606	−0.6	2.7	0.75	3.03
Env_exP	1.687879	0.6410291	−0.8	3.5	−0.38	4.36
**Logarithmic values**						
Variable	Mean	Std. Dev.	Minimum	Maximum		
logEnv_expT	5.794829	1.122908	2.525729	7.889759	1.28	3.90
logEnv_exp_GDPE	−0.4300593	0.4651294	−2.302585	0.3364722	0.14	2.57
logDGEXP	1.526407	0.2032495	1.006429	1.911018	−0.27	2.44
logEPEXPG	−1.985885	0.5391884	−2.302585	0.0953102	−0.19	2.28
logCHEXP	1.870832	0.1605441	1.432208	2.173861	−1.67	7.44
Env_protection_Texp	−1.339527	0.7362824	−2.302585	0.9932518	−0.25	2.33
logEnv_exP	0.4578629	0.4429094	−1.609438	1.252763	−1.95	8.28

Note: maximum, minimum, standard deviation, skewness, kurtosis for the total number of 198 observations and for logarithmic values

## References

[1] John A., Pecchenino R. (1994). An overlapping generations model of growth and the environment. Econ. J..

[2] Grossman G.M., Krueger A.B. (1995). Economic Growth and the Environment. Q. J. Econ..

[3] Byrne M.M. (1997). Is growth a dirty word? Pollution, abatement and endogenous growth. J. Dev. Econ..

[4] Bertinelli L., Strobl E., Zou B. (2008). Economic development and environmental quality: A reassessment in light of nature’s self-regeneration capacity. Ecol. Econ..

[5] Bostan I. (2016). Pro sustainable development: The influence of the law of entropy on economic systems. Environ. Eng. Manag. J..

[6] Condrea P., Bostan I. (2008). Environmental issues from an economic perspective. Environ. Eng. Manag. J..

[7] Bostan I., Burciu A., Condrea P. (2010). Trends of the communitarian cohesion policies and advertising for eco-investments. Environ. Eng. Manag. J..

[8] Burciu A., Bostan I., Condrea P., Grosu V. (2010). Financing the environmental policies in the Communitarian space. Environ. Eng. Manag. J..

[9] Angelopoulos K., Economides G., Phillipopoulos A. (2013). First- and second-best allocations under economic and environmental uncertainty. Int. Tax Public Financ..

[10] Kuo T.C., Hong I.H., Lin S.C. (2016). Do carbon taxes work? Analysis of government policies and enterprise strategies in equilibrium. J. Clean. Prod..

[11] Vasilev A. (2019). Optimal fiscal policy with environmental tax and pollution abatement spending in a model with utility-enhancing environmental quality: Lessons from Bulgaria. Macroecon. Financ. Emerg. Mark. Econ..

[12] Pohoață I., Bostan I., Prelipcean G., Druguș D., Morariu A., Bunget O. (2014). Equity, intra/inter-generation equalization and profit, in the context of the right to a healthy life and a clean environment. Rev. Rom. Bioet..

[13] Barman T.R., Gupta M.R. (2010). Public expenditure, environment, and economic growth. J. Public Econ. Theory.

[14] Adewuyi A.O. (2016). Effects of public and private expenditures on environmental pollution: A dynamic heterogeneous panel data analysis. Renew. Sustain. Energy Rev..

[15] Xie X., Wang Y. (2018). Evaluating the Efficacy of Government Spending on Air Pollution Control: A Case Study from Beijing. Int. J. Environ. Res. Public Health.

[16] Bostan I. (2015). Promotion and Support of the Natural Capital: Research on Ensuring the Financial Resources for the Conservation of Biodiversity (CBD) in the Romanian Space. Int. J. Environ. Res..

[17] Baumol W.J., Oates W.E. (1988). The Theory of Environmental Policy.

[18] Oueslati W. (2002). Environmental policy in an endogenous growth model with human capital and endogenous labour supply. Econ. Model..

[19] Fischer C., Heutel G. (2013). Environmental macroeconomics: Environmental policy, business cycles, and directed technical change. Annu. Rev. Resour. Econ..

[20] López R., Palacios A. (2014). Why Europe has become environmentally cleaner: De-composing the roles of fiscal, trade and environmental policies. Environ. Resour. Econ..

[21] Bostan I., Onofrei M., Dascălu E.D., Fîrțescu B., Toderașcu C. (2016). Impact of sustenaible environmental expenditures policy on air pollution reduction, during European integration framework. Amfiteatru Econ..

[22] Bostan I., Burciu A., Condrea P., Durac G. (2009). Involvement of legal responsibility for severe acts of pollution and noncompliance. Environ. Eng. Manag. J..

[23] IMF (International Monetary Fund) (2012). Fiscal Policy to Mitigate Climate Change: A Guide for Policymakers.

[24] IMF (2016). After Paris: Fiscal, Macroeconomic, and Financial Implications of Climate Change (IMF Staff Discussion Note SDN/16/01).

[25] EEA (European Environment Agency) (2011). Environmental Tax Reform in Europe: Implications for Income Distribution (Technical Report No. 16/2011).

[26] Dinda S. (2005). A theoretical basis for the environmental Kuznets curve. Ecol. Econ..

[27] Wilson J., Tyedmers P., Pelot R. (2007). Contrasting and comparing sustainable development indicator metrics. Ecol. Indic..

[28] Jouvet P.A., Michel P., Rotillon G. (2005). Optimal growth with pollution: How to use pollution permits?. J. Econ. Dyn. Control.

[29] Halkos G.E., Paizanos E.A. (2016). Environmental Macroeconomics: Economic Growth, Fiscal Spending and Environmental Quality. Int. Rev. Environ. Resour. Econ..

[30] D’Orazio P., Popoyan L. (2019). Fostering green investments and tackling climate-related financial risks: Which role for macroprudential policies?. Ecol. Econ..

[31] Economides G., Philippopoulos A. (2008). Growth enhancing policy is the means to sustain the environment. Rev. Econ. Dyn..

[32] Gupta M.R., Barman T.R. (2009). Fiscal policies, environmental pollution and economic growth. Econ. Model..

[33] Halkos G.E., Paizanos E.A. (2013). The effect of government expenditure on the environment: An empirical investigation. Ecol. Econ..

[34] Lopez R., Galinato G., Islam A. (2013). Fiscal Spending and the Environment: Theory and Empirics. J. Environ. Econ. Manag..

[35] Itaya J. (2008). Can environmental taxation stimulate growth? The role of indeterminacy in endogenous growth models with environmental externalities. J. Econ. Dyn. Control.

[36] Islam F., Lopez R. (2015). Government spending and air pollution in the U.S. Int. Rev. Environ. Resour. Econ..

[37] Wang S., Zhou D., Zhou P., Wang Q. (2011). CO2 emissions, energy consumption and economic growth in China: A panel data analysis. Energy Policy.

[38] Halkos G.E., Paizanos E.A. (2016). The effects of fiscal policy on CO2 emissions: Evidence from the U.S.A. Energy Policy.

[39] Rodríguez M.C., Dupont-Courtade L., Oueslati W. (2016). Air pollution and urban structure linkages: Evidence from European cities. Renew. Sustain. Energy Rev..

[40] Frankel J.A., Rose A.K. (2005). Is trade good or bad for the environment? Sorting out the causality. Rev. Econ. Stat..

[41] Khan M.A., Khan M.Z., Zaman K., Naz L. (2014). Global estimates of energy consumption and greenhouse gas emissions. Renew. Sustain. Energy Rev..

[42] Guan D., Hubacek K., Weber C., Peters G., Reiner D. (2008). The drivers of Chinese CO emissions from1980 to 2030. Glob. Environ. Chang..

[43] Gassebner M., Lamla M.J., Sturmz J. (2011). Determinants of pollution: What do we really know?. Oxf. Econ. Pap..

[44] World Economic Forum’s Global Leaders for Tomorrow Environment Task Force (WEF), Yale Center for Environmental Law and Policy (YCELP), Columbia University Center for International Earth Science Information Network (CIESIN) (2001). The Environmental Sustainability Index (ESI). http://www.ciesin.columbia.edu/indicators/ESI/.

[45] Wendling Z.A., Emerson J.W., Esty D.C., Levy M.A., De Sherbinin A. (2018). Environmental Performance Index.

[46] Stern C., Young R., Druckman D. (1992). Global Environmental Change: Understanding the Human Dimensions.

[47] Jedrychowski W. (1995). Review of recent studies from Central and Eastern Europe associating respiratory health effects with high levels of exposure to “traditional” air pollutants. Environ. Health Perspect..

[48] Pope C.A., Dockery D.V. (2013). Air pollution and life expectancy in China and beyond. Proc. Natl. Acad. Sci. USA.

[49] Pope C.A., Bates D.V., Raizenne M.E. (1995). Health effects of particulate air pollution: Time for reassessment?. Environ. Health Perspect..

[50] Patz J.A., Campbell-Lendrum D., Holloway T., Foley J.A. (2005). Impact of regional climate change on human health. Nature.

[51] Yazdi S.K., Khanalizadeh B. (2017). Air pollution, economic growth and health care expenditure. Econ. Res. Ekon. Istraživanja.

[52] Cakmak S., Burnett R.T., Krewski D. (1999). Methods for detecting and estimating population threshold concentrations for air pollution-related mortality with exposure measurement error. Risk Anal..

[53] Evans M.F., Smith V.K. (2005). Do new health conditions support mortality-air pollution effects?. J. Environ. Econ. Manag..

[54] Brunekreef B. (1997). Air pollution and life expectancy: Is there a relation?. Occup. Environ. Med..

[55] Mariani F., Pérez-Barahona A., Raffin N. (2009). Life Expectancy and the Environment. IZA Discussion Paper.

[56] Jouvet P., Pestieau P., Ponthiere G. (2010). Longevity and environmental quality in an OLG model. J. Econ..

[57] Atash F. (2007). The deterioration of urban environments in developing countries: Mitigating the air pollution crisis in Tehran, Iran. Cities.

[58] Tanner M., Harpham T. (2014). Urban Health in Developing Countries: Progress and Prospects.

[59] Jha R., Whalley J. (2015). The environmental regime in developing countries. World Scientific Reference on Asia and The World Economy.

[60] Dong Y., Hauschild M.Z. (2017). Indicators for environmental sustainability. Procedia CIRP.

[61] Tang L., Ii R., Tokimatsu K., Itsubo N. (2018). Development of human health damage factors related to CO2 emissions by considering future socioeconomic scenarios. Int. J. Life Cycle Assess..

[62] Rabl A., Eyre N. (1998). An estimate of regional and global O3 damage from precursor NOx and VOC emissions. Environ. Int..

[63] Logue J.M., Price P.N., Sherman M.H., Singer B.C. (2012). A method to estimate the chronic health impact of air pollutants in U.S. residences. Environ. Health Perspect..

[64] Fernando M., Samita S., Abeynayake R. (2012). Modified Factor Analysis to Construct Composite Indices: Illustration on Urbanization Index. Trop. Agric. Res..

[65] Wang X., Kammerer C.M., Anderson S., Lu J., Feingold E.A. (2009). Comparison of Principle Component Analysis and Factor Analysis Strategies for Uncovering Pleiotropic Factors. Genet. Epidemiol..

[66] Eck N.J., Waltman L. (2009). How to normalize cooccurrence data? An analysis of some well-known similarity measures. J. Am. Soc. Inf. Sci. Technol..

[67] Jha R., Murthy K.V. (2003). A Critique of the Environmental Sustainability Index.

[68] Siche J.R., Agostinho F., Ortega E., Romeiro A. (2008). Sustainability of nations by indices: Comparative study between environmental sustainability index, ecological footprint and the emergy performance indices. Ecol. Econ..

[69] Rogge N. (2012). Undesirable specialization in the construction of composite policy indicators: The Environmental Performance Index. Ecol. Indic..

[70] Hertz E., Hebert J.R., Landon I. (1994). Social and environmental factors and life expectancy, infant mortality, and maternal mortality rates: Results of a cross-national comparison. Soc. Sci. Med..

[71] Girum T., Muktar E., Shegaze M. (2018). Determinants of life expectancy in low and medium human development index countries. Med. Stud. Studia Med..

[72] McMichael A.J. (2009). Human population health: Sentinel criterion of environmental sustainability. Curr. Opin. Environ. Sustain..

[73] Tilman D., Clark M. (2014). Global diets link environmental sustainability and human health. Nature.

[74] Nixon J., Ulmann P. (2006). The relationship between health care expenditure and health outcomes. Eur. J. Health Econ..

[75] Pautrel X. (2007). Pollution, Health and Life Expectancy: How Environmental Policy Can Promote Growth. SSRN Electron. J..

[76] Ansari M.R., Mussida C. (2013). NORM: Stata Module to Normalize Variables.

[77] Liu J., Raven P.H. (2010). China’s environmental challenges and implications for the world. Crit. Rev. Environ. Sci. Technol..

[78] Šauer P., Švihlová D., Dvořák A., Lisa A. (2013). Visegrad Countries: Environmental Problems and Policies.

[79] Urbaniec M. (2014). Implementation of International Standards for Environmental Management in Visegrad Countries: A Comparative Analysis. Entrep. Bus. Econ. Rev..

